# A qualitative study of a psychiatric emergency

**DOI:** 10.1186/1752-4458-2-9

**Published:** 2008-06-30

**Authors:** Yves Chaput, Michel Paradis, Lucie Beaulieu, Édith Labonté

**Affiliations:** 1 Department of Psychiatry, McGill University, Montreal, Canada; 2 Department of Psychiatry, CHUM Hospital ('*Centre Hospitalier de l'Université de Montréal*'), Montreal, Canada; 3 Department of Psychiatry, Haut-Richelieu Hospital, Saint-Jean-sur-Richelieu, Quebec, Canada; 4 Department of Psychiatry, Enfant-Jesus Hospital, University Laval, Quebec city, Quebec, Canada

## Abstract

**Background:**

The psychiatric emergency service (PES) is a major hub in the mental health care delivery system. The aim of this study was to more precisely define what psychiatrists consider to be a psychiatric emergency and to examine the underlying basis of this assessment.

**Methods:**

Over twenty-two thousand PES visits were assessed prospectively for pertinence and urgency by psychiatrists in four functionally and structurally different services in the province of Quebec, Canada. This study took place between July 15 1996 and August 31, 2004.

**Results:**

Overall, 57% of visits were judged pertinent and urgent (P/U), 30% pertinent but not urgent (P/NU) and 13% neither pertinent nor urgent (NP/NU). Between 50 and 60% of P/U tagged visits were diagnosed with an affective or a psychotic disorder, often with a suicidal content. They also more frequently resulted in a short-term observation in the PES or a hospitalization. Variables suggesting the presence of a behaviorally disturbed state (aggressive behaviors, involuntary or police referrals) were equally likely to be found in P/U or NP/NU visits. Legal confinement following the consultation was almost exclusively seen in visits judged P/U. The percent of visits tagged P/U at the four individual sites varied substantially above and below the 57% value for the combined data. Interestingly, no major inter-site differences in diagnostic profiles for the three pertinence and urgency anchor points were found that might account for this variability. Finally, visits from high frequency users were less likely to be judged P/U than visits from patients attending less frequently.

**Conclusion:**

Primary consideration for a P/U tag was a visit characterized by a behaviorally disturbed state and/or, suicidal ideation (or attempts) within the context of either an underlying psychotic or affective disorder, especially if poor judgment was an issue. Some specific diagnoses appeared to qualify the above core clinical considerations, increasing or decreasing the probability of a P/U tag. Finally, non-clinical site-specific factors related to the individual services themselves, such as the number of readily available specialized resources, also appeared to qualify this assessment. These data may prove useful for the future development of this service.

## Background

The psychiatric emergency service (PES) is an important hub in the mental health care delivery system. In its most elementary form it consists in the interaction between a psychiatrist (or health care professional) and a patient within the framework of a crisis situation. Despite many published reports concerning this interaction the very nature of what constitutes a 'psychiatric emergency' remains ambiguous. The latter can be deduced, at least in part, from quantitative studies where the socio-demographic and clinical characteristics of PES patients are assessed [1-3]. These studies provide an increasingly detailed, diagnostic-specific picture of who visits this service.

A PES visit however can also include a more subjective, qualitative type of information that might also prove useful in defining a psychiatric emergency. For instance, quantitative studies have shown that over time most PES visits are made by patients who come repeatedly, some at a very high frequency [1-3], suggesting that a host of different reasons may be important in a patient's decision to seek immediate help. Conversely, physicians or health care professionals may view some, but not all, of these reasons as being an acceptable use of an expensive medical service. In a large, qualitative analysis of 1002 visits to a PES in Dallas, Texas [4] a substantial discrepancy was observed between what psychiatrists subjectively considered 'appropriate' PES use (70% of visits) and, visits graded by hospitalization as the outcome (20% of visits) or, graded for emergent illness (20% of visits) using traditional medico-surgical criteria. All three rating methods seemed to identify 'behavioral dyscontrol' and dangerousness as the best correlates of appropriate service use. Overall however, the combination of all three methods was more successful in determining what was not (30% of visits) rather that what was (8% of visits) a psychiatric emergency, adding to the consensus that up to 20% of PES visits involve non-psychiatric social or coping difficulties perhaps better managed by non-psychiatric community services [4-14].

Psychiatric decision-making in the PES strongly influences visit outcome and by default, costs. Typical inpatient stays for depression in Canada are comparable in costs to those for chronic obstructive pulmonary disease or heart failure [15]. Better defining what psychiatrists consider a pertinent and urgent referral (and the complex clinical and administrative factors that might underlie such a decision) could ultimately prove useful for service planning and development. This was the primary objective of this study and was pursued by using a database of over twenty-two thousand visits prospectively assessed for pertinence and urgency by psychiatrists in four PESs in the province of Quebec, Canada. As these services were functionally and structurally different they offered the added benefit of assessing how actual service organization might influence this decision making process.

## Methods

Clinical and demographic data were obtained from all adult patients visiting site A, the PES of a university teaching hospital in Montreal Canada from July 15, 1996, to June 15, 2004. Up to 70 variables per visit could be acquired, including three DSM-IV diagnoses. To reduce diagnostic uncertainty diagnoses were grouped into broad categories. Also acquired was a psychiatrist-rated subjective evaluation of the consultation process. Three anchor points were used; pertinent and urgent (P/U), pertinent but not urgent (P/NU), neither pertinent nor urgent (NP/NU) and, could not be determined, loosely based upon anchor points used in prior studies [7,16]. However, in accordance with Claassen et al., [4] the anchor points were largely left undefined, depending upon how psychiatrists interpreted them. Only rough guidelines were provided, such as equating pertinence with 'presence or absence of a psychiatric problem' and urgent with 'requires immediate attention' and not urgent with 'could have waited a week or more for an outpatient visit'.

Particular attention was paid to variables that might influence an anchor point assessment, such as those suggesting a state of behavioral disturbance. These included aggressive behavior (verbal, physical aggression or both), arrival by ambulance, police or involuntary referral to the PES (regardless of the original source of the referral) and legal confinement to the hospital following the psychiatric consultation. Also assessed were pharmacological variables such as percent of patients presently taking medication, the type of medication (antidepressants, oral anti-psychotics, intra-muscular anti-psychotics, hypnotics, benzodiazepines, mood stabilizers) and medication compliance. All data was collected prospectively. The database was used until December 31, 2000 and again from September 2002 to June 15, 2004 during the multi-site part of the study described below. A more detailed description of the database and of data acquisition has been provided in prior publications [2,17,18].

The data described above were also collected prospectively for a two-year period (beginning September 2002) for patients visiting three other services (sites B, C and D). Sites C and D were in cities other than Montreal (C was in a city of about one hundred thousand citizens located 30 km from Montreal whereas D was in Quebec city). All sites (other that site C where the coverage was partial) were covered by experienced psychiatric and nursing staff during the daytime, when most visits have been shown to occur [2]. Most psychiatric staff had over five years of experience in the PES setting. In addition, most obtained their medical and specialty training at one of the four universities in the province. They thus shared a common set of cultural/ethical standards. During night calls residents in psychiatry assessed newly arrived patients under supervision by staff and, guidelines obliged staff to be present during the interview for junior residents. Sites A, C and D were within general hospitals whereas site B was in a psychiatric institute. Therefore, patients visiting site B were not medically triaged prior to the psychiatric consultation. Site C, unlike the other sites, did not have a holding area. Holding areas (usually 8 beds and staffed by psychiatric personnel) were typically in a secure, closed area separate although adjacent to the medical emergency room. Observation periods were typically up to 96 hrs at sites A and B whereas site D limited most observations to 48 hrs. Site C, the only non-university hospital, had a brief therapy unit within the hospital permitting up to 14-day observation periods. Site D had 68% of visits assessed or co-assessed by psychiatric residents versus only 25%, 8% and 0% of visits at sites A, B and C, respectively. Although aver 40 staff members participated in this study, well over half of all visits were assessed by the regular 12 to 15 psychiatrists who specialized in the daily coverage of the four participating services.

### Primary data analysis

Data were analyzed by using the statistical analysis program Systat (Version 12). Nominal variables were analyzed using the 'crosstabulation' section for one- and two-way tables after their transformations into percentage tables. The four tables containing non-diagnostic variables present naturalistic data. For the two tables presenting diagnostic variables odds ratios (the independent variable being the P/U anchor points) were used as a measure of association between upper and lower cells (extracted as in 2 × 2 tables). Significance (all p values were < 0.05) was double-checked by examining the natural logarithm of the ratio and the standard error of the transformed ratio. Approximate 95% confidence intervals were constructed using the statistic plus or minus two times its standard error to ensure that a 0 value was not included in the intervals.

### IRB review

This study was approved by the institutional review board (IRB) scientific subcommittees at all sites and, other than at site B where full IRB approval was required, was exempted from full review.

## Results

### Combined results

All sites combined there were 26,311 visits where pertinence and urgency was assessed. Of these, 84% (N = 22,120) had ratings other than 'could not be determined' and are further analyzed below. Fifty seven percent of the 22,120 visits were judged P/U whereas 30% were judged P/NU and 13% were judged NP/NU.

Reasons for a psychiatric referral, available for 22,094 of the 22,120 above visits, were varied. For clarity, reasons that were closely related were collapsed into logical groupings. The only logical grouping frequently associated with a P/U tag (and progressively less so with the other two anchor points) was psychosis. In contrast, visits referred for anxiety or depression were more frequently attributed a P/NU tag. Some of the other logical groupings, such as suicide (attempts and ideation), although prevalent in P/U tagged visits, were also prevalent in the other two anchor points (Table 1).

**Table 1:** Pertinence/urgency and the reasons for a psychiatric referral^ab^.

	**None**	**Anx**.	**Psy**.	**Dep**.	**Sui**.	**DisBe**.	**Other 1**	**Other 2**	**Visits**
P/U	3%	7%	25%	18%	25%	9%	10%	4%	12495
P/NU	4%	15%	13%	22%	25%	8%	11%	3%	6654
NP/NU	7%	7%	11%	13%	32%	10%	17%	4%	2945

^a ^None (no given reason), Anx (anxiety, panic, agoraphobia, somatization, posttraumatic anxiety), Psy (hallucinations, delusional ideation, mania, psychosis, relapsed schizophrenia, disorganization), Dep (depression), Sui (suicidal ideation, suicide attempt), DisBe (disruptive behavior). Other 1 (anorexia, side-effects, homicidal ideation, hypomania, insomnia, intoxications, substance abuse, legal, psychiatric opinion, organicity), Other 2 (any reason other than those above).

^b ^The sum of each row may not add up to 100% due to rounding.

The diagnostic profiles for the three anchor points were next examined (Table 2, where a cell is fused if it does not differ significantly from the cell underneath, as assessed by Odds Ratios). Overall, affective and psychotic spectrum disorders comprised 55% of consultations judged P/U. In contrast, substance abuse disorders and visits where no discernable psychiatric diagnosis could be attributed comprised 66% of consultations judged NP/NU. We also examined the percentage distribution of P/U tagged visits within each individual broad diagnostic category. As expected, the psychosis not otherwise specified category had 90% of visits tagged P/U, versus 81% of bipolar, 68% of schizophrenia, 65% of depression, 53% of organic mental disorders, 51% each for personality and adjustment disorders, 40% of substance abuse and, 36% for the anxiety disorders category.

**Table 2:** The diagnostic^a ^profile, expressed as a % of the total number of visits^b ^for each anchor point.

	**None**	**Adj**	**Pd**	**Dep**	**Bipol**	**Sch**	**Anx**	**Sa**	**Pnos**	**Other**	**Visits**
P/U	3%	10%	13%	***18%***	10%	18%	3%	***12%***	9%	4%	**12511**
P/NU	5%	16%	17%		4%	13%	10%		2%	4%	**6658**
NP/NU	18%	5%	13%	1%	1%	7%	3%	48%	< 1%	5%	**2951**

^a^Adj (adjustment disorders), Pd (personality disorders), Dep (unipolardepression, dysthymia), Bipol (bipolar disorders), Sch (schizophrenia), Anx (anxiety disorders), Sa (substance abuse), Pnos (psychosis not otherwise specified), Other (sexual, eating, impulse control disorders, paranoid psychoses, organic mental disorders).

^b^The sum of each row may not add up to 100% due to rounding. Fused cells represent cells that do not differ significantly from each other, as assessed by ORs. A single value in a fused cell represents a value common to both anchor points. ORs were not calculated for the 'Other' category or for cells with a value of less than 1%.

Once an anchor point was assessed, psychiatrists were quite consistent regarding outcome. Outcome could be assessed in 20,429 of the combined 22,120 visits. Fully 34% of P/U visits resulted in a hospitalization (versus 3% for P/NU and 1% for NP/NU visits), 21% in an observation in the PES (versus 13% for P/NU and 12% for NP/NU visits) and 3% were transferred to another PES for an observation (1% for P/NU and NP/NU visits). Conversely, only 40% of P/U visits were discharged following the consultation compared to 80% and 73% of P/NU and NP/NU visits, respectively. Of note was the relatively large number 12% of NP/NU visits (2% for P/U and 4% for P/NU visits) who refused treatment, left without being formally discharged or were returned to the medical emergency department.

The particular constellation of diagnoses, reasons for a referral and outcomes in P/U tagged visits suggests the presence of some degree of 'behavioral dyscontrol'. Variables compatible with such a state were examined and were found to exhibit a distinct pattern of distribution. With the exception of legal confinement to the PES, they were usually equally present in P/U and NP/NU tagged visits and less so in the P/NU ones. For example, 10% of P/U and NP/NU visits exhibited aggressive behaviors versus 6% of P/NU visits (assessed in 22,119 of 22,120 visits). Arrival by ambulance was observed in 24% of P/U and 31% of NP/NU visits but only 17% of P/NU ones (assessed in 17,108 of 22,120 visits). Police referrals represented 12% of P/U and NP/NU visits but only 5% of P/NU ones (assessed in 17,108 of 22,120 visits). Involuntary referrals represented 22% of P/U and 19% NP/NU visits but only 8% of P/NU visits (assessed in 16,758 of 22,120 visits). Legal confinement to the PES following the evaluation was observed in 7% (N = 1339) of the 17,990 visits where this data was available. Most of these visits (94%, N = 1261) were tagged P/U. However, 4% (N = 52) and 2% (N = 26) of visits resulting in legal confinement were tagged P/NU or NP/NU, respectively. With regards to all pharmacological variables described in the methods section none showed any consistent or marked differences between the anchor points for the combined (or site-specific) data.

### Site-specific results

Site-specific results as well as site characteristics are presented in Table 3. Individual sites demonstrated substantial variability above and below the 57% of visits tagged P/U for the combined results. We examined whether at least part of this variability could be attributed to different diagnostic profiles per anchor point at the different sites (Table 4). That at site A most closely resembled the combined data in terms of significance between adjacent cells and individual cell percentages. The diagnostic profiles per anchor point at sites B, C and D showed a bit more variance. Notable was a proportional increase in depressive disorders tagged P/U at site C at and a high percentage of 'no diagnosis' (typically tagged NP/NU at all sites) at site B. Although these two findings could have contributed to the overall higher number P/U tagged visits at site C and the lower number of P/U visits at site B, they were nevertheless relatively minor differences across sites that had largely similar diagnostic profiles per anchor point.

**Table 3:** Visit pertinence and urgency per site and site characteristics.

	P/U	P/NU	NP/NU	Visits	DH^a^	1^st ^E^b^	S&P^c^	ACT^d^	PBD^e^	CT^f^	CC^g^	DC^h^	ES^i^
A	42%	35%	22%	7,324	1	N	N	N	N	Y	Y	≥ 5	≥ 5
B	34%	53%	13%	5,407	3	Y	Y	Y	Y	Y	Y	≥ 5	≥ 5
C	78%	15%	6%	3,109	1	N	N	Y	N	Y	N	< 5	< 5
D	81%	12%	7%	6,280	1	N	N	Y	Y	N	N	≥ 5	≤ 5

^a ^DH, the number of day hospitals the PES has access to.

^b ^1^st ^E, a first episode psychosis team that rapidly evaluates patients while in the PES.

^c ^S&P, a severe and persistent psychosis treatment team that evaluates patients while in the PES.

^d ^ACT, assertive community treatment team that evaluates patients while in the PES.

^e ^PBD, a specialized outpatient clinic for severe borderline personality disorders in the hospital.

^f ^CT, an in hospital crisis team that rapidly (7 to 14 days) evaluate patients referred from the PES.

^g ^CC, community based crisis center with inpatient beds that rapidly evaluate patients while still in the PES.

^h ^DC, the number of and, access to, substance abuse treatment centers.

^i ^ES, the number of emergency shelters for short (days) to medium (months) term stays.

**Table 4:** The diagnostic^a ^profile, expressed as a % of the total number of visits^b ^for each anchor point.

		**None**	**Adj**	**Pd**	**Dep**	**Bipol**	**Sch**	**Anx**	**Sa**	**Pnos**	**Other**	**Visits**
	P/U	5%	8%	13%	15%	10%	20%	3%	***12%***	10%	4%	3112
			
A	P/NU	2%	19%	17%	17%	2%	14%	10%	***13%***	2%	4%	2583
	
	NP/NU	12%	3%	13%	1%	1%	8%	2%	57%	< 1%	3%	1629

	P/U	4%	9%	9%	***21%***	11%	20%	6%	6%	9%	6%	1860
			
B	P/NU	7%	14%	16%	***19%***	4%	12%	12%	10%	1%	5%	2859
	
	NP/NU	37%	4%	9%	1%	2%	6%	6%	30%	<1%	5%	688

	P/U	2%	11%	***13%***	28%	11%	***15%***	3%	***9%***	6%	3%	2439
							
C	P/NU	4%	***16%***	***12%***	23%	7%	***12%***	8%	***12%***	2%	5%	469
			
	NP/NU	8%	***10%***	***17%***	5%	1%	4%	1%	48%	< 1%	4%	201

	P/U	***3%***	**11%**	13%	***15%***	10%	***16%***	3%	***15%***	9%	4%	5100
							
D	P/NU	***4%***	**15%**	**21%**	***13%***	6%	***11%***	7%	***16%***	3%	5%	747
			
	NP/NU	11%	7%	**18%**	2%	1%	3%	2%	46%	< 1%	8%	433

^a^Adj (adjustment disorders), Pd (personality disorders), Dep (unipolardepression, dysthymia), Bipol (bipolar disorders), Sch (schizophrenia), Anx (anxiety disorders), Sa (substance abuse), Pnos (psychosis not otherwise specified), Other (sexual, eating, impulse control disorders, paranoid psychoses, organic mental disorders).

^b^The sum of each row may not add up to 100% due to rounding. Bold/italic cells represent cells that do not differ significantly from each other, as assessed by ORs. ORs were not calculated for the 'Other' category or for cells with a value of less than 1%.

As was the case with the combined data, one assessed, a given anchor point predicted outcome. At all sites, P/U tagged visits more likely resulted in an observation or a hospitalization whereas NP/NU tagged visits, with the exception of a 20% observation rate at site D, were overwhelmingly discharged from the PES (Table 5). Reasons for a psychiatric referral were next examined (Table 6). As expected, site B, operating as a walk-in clinic, had a profile that differed most from that of the combined data and appeared weighted towards reasons pertaining to anxiety. That site B is a major specialized treatment center for eating disorders is also reflected in the data. Otherwise, reasons pertaining to psychosis, depression or suicide (ideation or attempts) comprised 78% of all P/U tagged visits at site A and 65% at sites C and D whereas site B had the lowest proportion (60%). As was the case with the combined data, of the above three reasons, those pertaining to psychosis appeared to be the most specific to a P/U tag at all sites (Table 6). Regarding variables compatible with a state of 'behavioral dyscontrol' (aggressive behaviors, police referral or arrival by ambulance, involuntary arrivals) the specific sites data resembled the main finding (combined data) of being as prevalent in visits tagged P/U or NP/NU and less so in those tagged P/NU (data not shown). As for legal confinement, 3% (93 of 3196 visits), 5% (247 of 5405 visits), 6% (198 of 3109 visits) and 13% (801 of 6279 visits) of visits at sites A, B, C and D, respectively, resulted in legal confinement. The overwhelming majority of these were tagged P/U, although sites A and C were the most specific (92 of 93 cases and 194 of 198 who were placed in legal confinement were tagged P/U, respectively).

**Table 5:** Pertinence/urgency and visit outcome^ab ^per site.

	**Dis**.	**Obs**.	**Hosp**.	**Trans**.	**Other**	**Visits**
A- P/U	30%	36%	21%	7%	6%	3112
P/NU	75%	17%	1%	1%	7%	2583
NP/NU	72%	14%	1%	1%	11%	1629

B- P/U	52%	23%	23%	2%	< 1%	1777
P/NU	88%	7%	2%	1%	2%	2566
NP/NU	71%	8%	1%	< 1%	20%	626

C- P/U	41%	13%	42%	< 1%	2%	2311
P/NU	68%	17%	13%	< 1%	2%	424
NP/NU	77%	12%	4%	1%	5%	177

D- P/U	29%	18%	52%	1%	1%	4409
P/NU	68%	17%	13%	1%	1%	502
NP/NU	72%	20%	4%	2%	1%	313

^a ^Dis (discharge), Obs (observation), Hosp (hospitalization), Trans (transfer to another PES), Other (refused treatment, left after being seen without formal discharge or was returned to the medical emergency department).

^b ^The sum of each row may not add up to 100% due to rounding. Outcome was available for 20,429 of the 22,120 visits.

**Table 6:** Pertinence/urgency and the reasons for a psychiatric referral^ab ^per site.

	**None**	**Anx**.	**Psy**.	**Dep**.	**Sui**.	**DisBe**.	**Other 1**	**Other 2**	**Visits**
A- P/U	3%	4%	32%	17%	29%	7%	6%	2%	3110
P/NU	2%	9%	16%	25%	38%	6%	3%	1%	2583
NP/NU	8%	4%	14%	14%	40%	7%	12%	1%	1626

B- P/U	5%	12%	23%	17%	20%	12%	9%	1%	1851
P/NU	6%	20%	9%	22%	17%	10%	13%	3%	2857
NP/NU	10%	14%	7%	10%	14%	11%	29%	5%	687

C- P/U	4%	6%	22%	22%	21%	7%	11%	6%	2436
P/NU	4%	11%	16%	30%	14%	4%	15%	6%	467
NP/NU	5%	4%	10%	18%	26%	11%	17%	10%	199

D- P/U	2%	7%	21%	17%	27%	10%	11%	5%	5098
P/NU	3%	16%	11%	23%	17%	9%	12%	9%	747
NP/NU	2%	6%	7%	13%	32%	16%	15%	9%	433

^a ^See Table 2 for the list of the abbreviations.

^b ^The sum of each row may not add up to 100% due to rounding. Reasons were available for 22,094 of the 22,120 visits.

### Pertinence/urgency and frequent PES use

Lastly, the relationship between frequent service use and the three anchor points was examined. Previous reports have shown that patients making 11 or more visits at site A required approximately 9 years to complete all of their visits [2,18]. This time frame is much longer than the data acquisition period for the multi-site trial. Only data for site A, which spanned a full 8 years, was thus analyzed. Visits were divided into those made by patients with 1 to 3 visits (N = 4721 visits), 4 to 10 visits (N = 1317) or, 11 or more visits (N = 1283). The first two groups (those with 1 to 3 and those with 4 to 10 visits) had almost identical profiles. Overall, 44% of their visits were tagged P/U, 35% were tagged P/NU and 20% NP/NU. Visits from patients making 11 or more visits were almost equally likely to be tagged P/U (35%), P/NU (33%) or NP/NU (32%).

## Discussion

Our results suggest that several factors underlie what physicians consider to be a pertinent and urgent use of the PES. First and foremost would appear to be clinical considerations. In a generic sense, those presenting with what can be termed 'behavioral dyscontrol' and/or, suicidal ideation (or attempts) within the context of either an underlying psychotic or affective disorder, constitute what most psychiatrists in this study (and at all sites) consider a P/U visit. Moreover, these results were arrived at by consensus as the anchor points were largely left undefined. In some cases, the behavioral disturbance or suicidal aspect of the visit was sufficiently worrisome to warrant legal confinement to the service, suggesting that clinical factors such as poor judgment are also integrated into this assessment. Overall, these data are in agreement with previous reports suggesting that 'behavioral dyscontrol' or dangerousness are accurate indexes of both visit appropriateness and hospitalization as visit outcome [4,19].

Second, our results also suggest that there are qualifying factors to the above core clinical considerations. One is the qualifying effect of a specific diagnosis on the perceived pertinence of a consultation. Indeed, some variables, by themselves, did not appear to be particularly useful in the absence of a diagnostic profile. For instances, variables such as referral by police, by ambulance, aggressive behaviors or involuntary referrals were as frequently seen in NP/NU visits as those tagged P/U (combined and site-specific data). Behavioral disturbance or suicidal ideation (or attempts) due to alcohol or substance abuse or, to a lesser extent, due to a severe personality disorder, were often tagged NP/NU. This appeared to be so even when controlling for frequency of PES use (data not shown). That disorders like substance abuse might so qualify these variables might at first appear pejorative and must be put into context. There is debate in Canada as to who should treat these disorders [20,21] and publicly funded substance abuse treatment centers, some with a mental health component, are numerous in the greater Montreal region. The relatively low attribution of a P/U tag may reflect the notion that some of these patients could be directed towards these, rather than the more costly medical resources. It may also reflect the notion that suicidal ideation is present in almost 50% of alcohol abusers and that alcohol-related phenomena that might promote suicidal ideation (aggression and impulsivity) may subside substantially with the level of intoxication [22].

Third, a possible non-clinical qualifying factor to a pertinence and urgency assessment may be the functional and structural organization of the PES sites themselves. With the exception of a few differences in the diagnostic profiles of the three anchor points at certain sites (a greater relative proportion of affective disorders tagged P/U at site C, and a high relative proportion of NP/NU visits with no actual diagnosis at site B) the profiles were quite similar. The sum of the three broad diagnostic categories where one is likely to find a P/U tag (psychosis not otherwise specified, bipolar and schizophrenia) shows a very narrow range between sites (40% at sites A and B, 35% at D et 32% at site C).

Similar profiles per anchor point in the face of substantially varying visit totals for the overall P/U tag between sites suggest that, for each individual broad diagnostic category, the same patient tagged P/NU at one site is consistently being tagged P/U at another. Tables 2 and 4 illustrate this quite well. The number of visits in the 'visits' column of Table 2 (combined data) approximately double at each anchor point NP/NU (2,951), P/NU (6,658) and P/U (12,511). Although those in Table 4 also approximately double between the NP/NU and P/NU assessments for sites C and D, they increase by five to six-fold (rather than two-fold) thereafter at these same sites. The P/NU patients in each diagnostic category at sites C and D are shifted up to a P/U tag, leaving the relative contribution of each diagnostic category to the overall P/U profile little changed. This suggests that dangerousness and poor judgment excluded, a different type of risk assessment strategy is used at the different sites despite being staffed by equivalently trained physicians. Once assessed, however, all were consistent with regards to outcome inasmuch as P/U visits more frequently resulted in either hospitalization or short-term observation.

It would thus appear that factors specific to a given PES come into play while making a P/U assessment. Although one may speculate as to what these factors may be, an extensive array of community and medically based resources readily available to the PES would certainly be primary candidates (see Table 3). Physicians at site B, with rapid access to first episode psychosis, severe and persistent and ACT teams may have felt comfortable with a P/NU tag whereas the same patient would have been tagged P/U at sites C or D. In addition, the value of prior medical triage appears to be illustrated by the lower number of NP/NU tagged visits at sites other than B, which operates as a walk-in clinic.

More speculative, structural factors such as a brief therapy unit (site C) permitting much longer observation periods may reduce the outward looking vision helpful for maximum use of all community resources. Conversely, too rigid a time constraint in an observation unit (site D) combined with a high proportion of visits co-assessed by residents may exaggerate the number of P/U tagged visits. Studies have shown that PES hospitalization rates are at least partly correlated with the staff's clinical experience (see [8] for review). In this same light, continuity of care may also be important. Site A was the only site where staff was stable during the week and specialized in either reassessing patients under observation or assessing the incoming referrals. In contrast, site C alternated psychiatric staff on a daily basis and had less complete weekday coverage.

Precedence can be found for the considerable inter-site range (34 to 81%) of pertinent and urgent consultations reported here. In previous studies of prospectively rated PES visits Claassen et al., [4] in Dallas found that 70% of 1002 visits to an inner-city service were 'necessary'. Vigiser et al., [16] in Petah Tiqva (Israel) and MacKenzie and Mackie [7] in Edinburgh (Scotland) rated about 30% (N = 177 and N = 77, respectively) of general hospital PES visits as both urgent and justifiable. In the former study the diagnostic profiles underlying appropriate PES use were, with the exception of the absence of substance abuse, similar to those of the present study. Additionally, in analogy to site B, Mackenzie and Mackie [7] reported that self-referrals were much less likely than GP referrals to be attributed an urgent tag.

Finally, these data also shed some qualitative light on visits made by frequent users. Contrary to what would have been expected from the diagnostic profile of high frequency users [2,18] patients with a high number of visits were less likely to be attributed a P/U tag. Sixty-five percent of visits made by patients with 11 or more visits were not tagged P/U, suggesting that more often than not, these visits were not characterized by a highly disturbed state. That many high frequency users at these sites suffer from chronic schizophrenia and many were (or had been) under active multidisciplinary case management [2,18] further underscores the fact that repeat visits are a complex, multi-factorial phenomenon.

## Conclusion

In an era of budgetary restraints clinical services are called upon to better define and to better target their core functions in order to maintain (or increase) quality of care. This study offers a highly filtered view of what constitutes the core clientele of a PES, as seen through the eyes of PES psychiatrists. The other side of this equation, why patients actually come to the PES, was not examined and this constitutes one of this study's major limitations.

Core behavioral disturbances as well as diagnoses were found to be important clinical variables in assessing pertinence and urgency. Ideally, this should have sufficed and only minor inter-site variations should have been found. This was not the case. A pertinence and urgency assessment also appeared to include a complex web of site-specific functional and structural PES factors. This constitutes another major limitation for this study and limits how our data can be generalized. Sites were chosen because they differed significantly one from the other but possessed equivalently trained staff. Other PES models, not represented here, also exist. Therefore, although the PES-specific factors examined here may readily transcend the different mental health care delivery systems in use today, other factors may also be important. Our data nevertheless offers potential choices of building blocks for those interested in the future development of the PES. They range from models primarily based upon a limited number of functions judged to be medically pertinent to models that include many of the roles and functions now typically associated with the term 'mental health'. Our data also highlight several potential strategies that might be considered in order to increase both quality of care and cost effectiveness, regardless of the nature of the underlying model.

## List of abbreviations

PES: Psychiatric emergency service; OR: Odds Ratio; P/U: Pertinent and urgent; P/NU: Pertinent but not urgent; NP/NU: Neither pertinent nor urgent; ACT: Assertive community treatment.

## Declaration of competing interests

The authors declare that they have no competing interests.

## Authors' contributions

As primary author for this article I (Dr Yves Chaput) take full responsibility for the design of the trial, for data acquisition, data analysis and, data interpretation and the writing of the manuscript.

Dr Michel Paradis, Dr Lucie Beaulieu and Dr Édith Labonté were site principal investigators. They had significant input as to the design of the database and had full responsibility for the actual implementation of the study at their sites. They also had significant input as to the interpretation of the results.
